# Progress in Medicine

**Published:** 1896-05-09

**Authors:** 


### Progress in Medicine.

### DIABETES.

(*Continued from page* 75.)

**Bronzed Diabetes,** a term applied in 1886 to a form of diabetes, by Professor Hanot,[6] in which two morbid conditions are present, namely, diabetes and hypertrophic cirrhosis with pigmentation, a combination he described in 1882 in conjunction with Chauffard. In his second series of cases the liver was not appreciably enlarged, the normal volume and pigmentation being fully maintained. Chauffard agreed with him in considering the pigmentary cirrhosis as a consequence of the diabetes, and he admitted that under the influence of the glycosuria caused by the original condition and of the obstructed circulation due to diabetic endarteritis the hepatic cell became stimulated with regard to its chromatogenic functions, and became the seat of a pigmentary hypergenesis, the pigment being generated in the liver became diffused in the form of minute emboli throughout the whole organism. In reviewing the different opinions expressed on the pathology of bronzed diabetes by various authorities since Hanot first described the disease, he remarks that Letulle does not admit the hypergenetic action of the liver cells, as those which are most pigmented are the dead ones. He considers that they store it rather than form it, while he does not regard the pigment found in other tissues as originating in the liver, but as formed *in situ*, owing to a reduction of hæmoglobin. Brault and Galliard consider that the pigmentation occurs in consequence of the cirrhosis, and is due to the fact that the altered hepatic cell cannot elaborate and utilise the blood pigment presented to it, which Gonzolez attributes to a purely mechanical influence of the pigment deposit. The evolution of the malady, Mosés considers that the diabetes causes a change in the blood which produces the pigmentation, the sugar and pigments causing changes in blood vessels eventuating in cirrhosis. Marie believes that *diabète bronzé* is neither a pigmentary cirrhosis in diabetes nor a cachexie bronzée, but a true pathological entity; in fact, a new disease, and Hanot agrees with him. It is a vascular, not a biliary cirrhosis, the biliary ducts are, as a rule, unaffected, the spleen is enlarged, and the intestinal glands black. The ochre-coloured pigment which infiltrates most of the organs is an iron-containing pigment (reaction with ammonium hydric sulphate and potassium ferrocyanide), while Brault states that the melanin pigment and the pigment of Addison's disease have no reaction for iron. The symptoms consist in the general signs of diabetes, with abundant glycosuria, melanderma most marked upon the face, limbs, and genital organs, hypertrophic cirrhosis, with slight ascites. The onset being frequently sudden, death supervenes either during coma or from septic pneumonia following diabetic gangrene.

**Acetonuria and Coma.**—Hirschfeld's[7] observations show that acetone exists under normal conditions in the urine of a healthy person, but only when carbohydrates are excluded from the diet, excretion of acetone then reaching from 20 to 70 c.gr. daily, being small in amount at first, gradually increasing, and reaching the stationary point. In rich proteid diet the acetonuria is less than in moderate proteid diet, but whether the needs of nutrition are met by a liberal supply of fat or not, and during periods of fasting, the amount of acetone discharged remains uninfluenced, and therefore its excretion is not dependent on the decomposition of proteid bodies. When acetone has made its appearance as a result of the suppression of carbo-hydrates in the diet, it suffices to add from 50 to 100 grammes of such substances to make the acetonuria disappear within two to four days.

Among carbohydrates which exert the greatest influence upon acetonuria are starch, cane sugar, grape sugar, milk sugar, and mannite. Glycerine appears to be very active in this direction, whereas alcohol has no such influence, nor have salicylate of soda, or antipyrin. In addition to physiological and diabetic acetonuria, it manifests itself also in the course of affections of the stomach, and in fever, as well as in patients suffering from cancer. In all these conditions, however, the etiology of the acetonuria is the same, as it is due to the limitation of carbohydrates in the diet, for as a matter of fact Hirschfeld found that acetonuria supervening in the course of pnuemonia, typhus, scarlatina, gout, &c., decreases or disappears entirely if from 60 to 90 grammes of grape sugar, for instance, be administered daily.

We may further refer to Rosenfeld's[8] observations on acetonuria and its treatment. Along with Friedlander, Ephraim, Kobrak, and Honigmann he conducted experiments on physiological acetonuria and found that on an ordinary mixed diet the healthy individual excretes minute quantities of acetone; in the fasting states the amount of acetone is increased; yet, although with moderate quantities of proteid food acetonuria is likewise increased, with large quantities of proteid food it is not markedly increased; and further, marked acetonuria resulting from exclusive meat diet or fasting can be made to disappear after one or two carbohydrate meals. Acetonuria, he says, appears to be a function of moderate albuminous decomposition. Carbohydrate metabolism, [as well as an increase beyond a certain point of albuminous decomposition, lessens acetonuria. Regarding diabetic acetonuria he finds that it is earlier in appearance, and more pronounced than in health, and aceto-acetic acid is also present, the latter being rarely present in health. Acetonuria attending the use of a mixed diet is a different matter. If a diabetic excrete acetone in large amount when abundant carbohydrate is being supplied, it is pathological and peculiar to himself, the carbohydrate metabolism being defective or wanting, and hence the acetonuria. There are two important clinical facts—(1) That diabetic coma hardly ever occurs without acetonuria; and (2) that after the commencement of the proteid diet acetonuria on the one hand, and coma on the other, may occur; thus the least possible excretion of acetone should be aimed at in these cases. In severe cases feeding with as much proteid food as will produce 30 to 35 grammes of N. may be difficult, and the acetonuria increase, and thus there only remains the last resource of feeding with the smallest amount of albuminous food and with as rich as possible a carbohydrate supply, such as lœculose, cane-sugar, or with glycerine. He has twice succeeded in overcoming commencing coma by forced carbohydrate feeding.

**A Non-saccharine Preparation of Milk** for diabetic patients, suggested by Sidney Ringer,[9] may be sometimes useful, though, as we have shown, milk sugar is a very good form of administering the necessary carbohydrate to diabetics. The following is the method: Add to a pint and a half of milk about 90 c.cm. of a 10 per cent. solution of acetic acid. This precipitates a curd-caseinogen. It should be allowed to settle, and the clear fluid siphoned off and distilled water added. After settling, this should be decanted or siphoned off, and the curd should be filtered and well washed with distilled water. If it is then rubbed up in a mortar with some calcium carbonate, and water is added, and all the caseinogen becomes dissolved, the calcium carbonate soon settles and the milky fluid can be decanted off. The dissolved caseinogen behaves just like milk. If rennet and a calcium salt is added, and the mixture is heated to 40 deg. C., it quickly clots, the caseinogen becomes changed into casein, which precipitates by combining with the calcium salts. Glycerine, 2 per cent., may be added to make it more palatable.

J. A. M. Anders[10] records a case of diabetes in a girl aged nine years. The urine contained 1 per cent. of sugar. When placed on a strict diabetic diet the general condition became worse and debility more marked, although the urine was free from sugar. The patient was therefore allowed three ounces of starches daily, and very soon the appearance and behaviour of the child furnished incontestable proof of marked general betterment, notwithstanding that sugar appeared in the urine to the extent of five-tenths per cent.

[6] Brit. Med. Journ., Jan. 25, 1896. [7] Zeitsch. f. klin. Med. xxviii., Nos. 2-3, pp. 17-209; N. York. Amer. Med. Surg. Bull., Jan. 11, 1896, and Med. Week, Oct. 18. [8] Centr. f. inn. Med., Dec. 21, 1895; Brit. Med. Jour., Jan. 18, 1896, [9] Brit. Med. Journ., Dec. 7, 1895. [10] Int. Clinics, iv., No. 3. 1895.

